# Racial differences in phenotypic frailty assessment among general thoracic surgery patients

**DOI:** 10.1016/j.xjon.2023.10.001

**Published:** 2023-11-07

**Authors:** Johnathan R. Kent, Emily M. Silver, Rachel Nordgren, Arianna Edobor, David Fenton, Savanna Kerstiens, Daniel Rubin, Lauren J. Gleason, Justine Landi, Megan Huisingh-Scheetz, Darren S. Bryan, Mark K. Ferguson, Jessica S. Donington, Maria Lucia L. Madariaga

**Affiliations:** a Section of Thoracic Surgery, Department of Surgery, University of Chicago, Chicago, Ill; b Department of Psychology, University of Chicago, Chicago, Ill; c Department of Public Health Sciences, University of Chicago, Chicago, Ill; d Department of Anesthesia and Critical Care, University of Chicago, Chicago, Ill; e Section of Geriatric & Palliative Medicine, Department of Medicine, University of Chicago, Chicago, Ill

**Keywords:** frailty, preoperative evaluation, race

## Abstract

**Objectives:**

The American Association for Thoracic Surgery recommends using frailty assessments to identify patients at higher risk of perioperative morbidity and mortality. We evaluated what patient factors are associated with frailty in a thoracic surgery patient population.

**Methods:**

New patients aged more than 50 years who were evaluated in a thoracic surgery clinic underwent routine frailty screening with a modified Fried's Frailty Phenotype. Differences in demographics and comorbid conditions among frailty status groups were assessed with chi-square and Student *t* tests. Logistic regressions performed with binomial distribution assessed the association of demographic and clinical characteristics with nonfrail, frail, prefrail, and any frailty (prefrail/frail) status.

**Results:**

The study population included 317 patients screened over 19 months. Of patients screened, 198 (62.5%) were frail or prefrail. Frail patients undergoing thoracic surgery were older, were more likely single or never married, had lower median income, and had lower percent predicted diffusion capacity of the lungs for carbon monoxide and forced expiratory volume during 1 second (all *P* < .05). More non-Hispanic Black patients were frail and prefrail compared with non-Hispanic White patients (*P* = .003) and were more likely to score at least 1 point on Fried's Frailty Phenotype (adjusted odds ratio, 3.77; *P* = .02) when controlling for age, sex, number of comorbidities, median income, diffusion capacity of the lungs for carbon monoxide, and forced expiratory volume during 1 second. Non-Hispanic Black patients were more likely than non-Hispanic White patients to score points for slow gait and low activity (both *P* < .05).

**Conclusions:**

Non-Hispanic Black patients undergoing thoracic surgery are more likely to score as frail or prefrail than non-Hispanic White patients. This disparity stems from differences in activity and gait speed. Frailty tools should be examined for factors contributing to this disparity, including bias.

## Graphical Abstract

**Figure:**
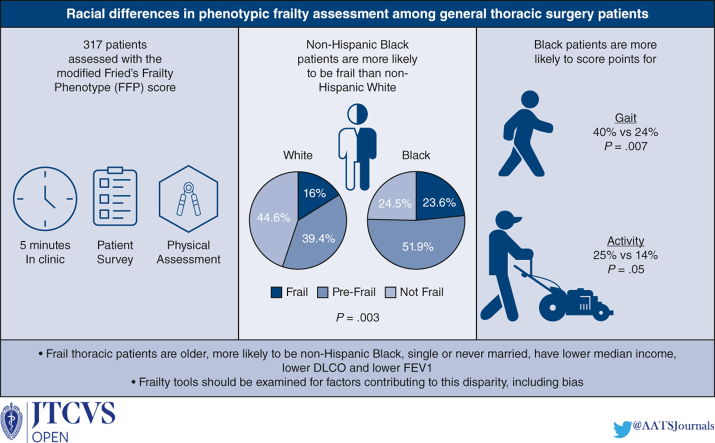

**Figure:**
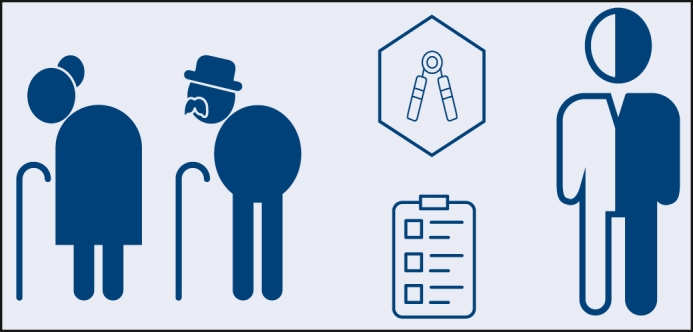
NHB patients are more likely to be frail than NHW patients.

**Central Message**

NHB patients undergoing thoracic surgery are more likely to score as prefrail or frail. Frailty tools should be examined for factors contributing to this disparity, including bias.

**Perspective**

We reviewed factors associated with frailty determined by FFP. Frail patients undergoing thoracic surgery were older, were more likely single, had lower median income, and had lower percent predicted DLCO and FEV1. Racial differences were also evaluated, with NHB patients more likely to be frail than NHW patients, driven by differences in activity level and gait speed.

Frailty is an age-associated clinical syndrome defined by limited physiologic reserve and increased vulnerability to stressors.^1^ It is estimated that 70% of patients currently seen by thoracic surgeons are prefrail or frail,^2^ a proportion that is expected to increase with an aging adult patient population.^3^ Frailty is associated with increased risk of postoperative complications, nonhome discharge, and higher mortality after surgery.^4^, ^5^, ^6^, ^7^ Identifying frailty status can inform surgeons about an individual's operative risk and encourage more personalized discussions during informed consent.^2^^,^^8^^,^^9^ The American Association for Thoracic Surgery highlighted frailty as an important factor in surgeon perioperative risk assessment in a recent consensus statement on high-risk patients for resection of stage I non–small cell lung cancer.^9^ The Society of Thoracic Surgeons recently established a Frailty Taskforce to incorporate a frailty screening metric into the Society of Thoracic Surgeons Database.

There is significant variability in assessment for frailty with more than 67 frailty assessment tools reported in the literature.^10^ Our group assesses frailty among patients undergoing thoracic surgery by the most frequently cited metric, Fried's Frailty Phenotype (FFP),^10^^,^^11^ which uses a combination of patient-provided subjective data (exhaustion, shrinkage, and activity level) with objective assessment (gait speed and grip strength), without relying on patient comorbidity burden.^1^ FFP is the frailty metric used in 25% of all frailty studies within cardiac surgery, and 1 of its components, gait speed, is used separately as a common stand-alone metric in evaluating patient perioperative risk.^12^^,^^13^

Frailty as identified by FFP has been associated with many sociodemographic and medical risk factors. Medical comorbidities including diabetes,^14^ history of cancer,^14^ heart failure, decreased forced expiratory volume in 1 second (FEV_1_), and functional vital capacity^15^, ^16^, ^17^ are associated with increased rates of frailty. Increased incidence of frailty is similarly associated with advanced age,^18^ female sex,^19^ poor social networks,^18^ lower income,^18^^,^^20^ and lower educational status.^18^^,^^20^ Furthermore, disparate scores on frailty assessments have been associated with race/ethnicity, with higher frailty incidence found in non-Hispanic Black (NHB) patients compared with non-Hispanic White (NHW) patients.^21^, ^22^, ^23^

We evaluate the association of FFP with patient factors within a thoracic surgery population. In doing so, we investigate the association of race with FFP and whether components of FFP vary by race. Identifying racial differences in frailty assessments of patients undergoing thoracic surgery may provide opportunities to target perioperative resources and mitigate disparities during surgical evaluation and treatment.^24^^,^^25^

## Material and Methods

### Patients

Routine frailty screening was instituted at the University of Chicago in December 2020 for new patients aged 50 years or older evaluated in the general thoracic surgery clinic. Patients in the current study were identified in a retrospective review of patients who were screened for frailty from December 2020 to June 2022. Demographic data including age, sex, body mass index (BMI), race/ethnicity, marital status, ZIP code of residence, and pulmonary function data (FEV1 and diffusion capacity of the lungs for carbon monoxide [DLCO] as percent predicted) were obtained on chart review. Comorbidity burden was evaluated for each patient, including diabetes mellitus, hypertension, cardiovascular disease (coronary artery disease, history of myocardial infarction, congestive heart failure), history of deep vein thrombosis, pulmonary disease (asthma, chronic obstructive pulmonary disease), arthritis, chronic kidney disease, and history of cancer. Overall comorbidity burden was categorized into patients without comorbidities, those with 1 to 2 comorbidities, and those with 3 or more comorbidities. Median income was derived from ZIP code of residence according to publicly available 2016-2020 census data, and then binned into quartiles.^26^ This study (IRB 21-1453) was reviewed by the University of Chicago Institutional Review Board and approved on December 17, 2021. Patient written consent for the publication of the study data was waived by the Institutional Review Board because some of the retrospective participants could be impossible to consent due to likelihood of patient death, loss to follow-up, change of care to other institutions, and other barriers.

### Frailty Assessment

Frailty was assessed using previously published updated FFP criteria used in the Successful Aging and Frailty Evaluation clinic by geriatricians at the University of Chicago (Table 1).^1^^,^^11^ Shrinking criteria were updated to include patients losing more than 5% of their prior body weight and patients who have a BMI less than 18.5. Recorded activities criteria were reduced from 17 (based on the Minnesota Leisure Time Activity Questionnaire) to 6 (based on Eckel's validated modification).^27^^,^^28^ The 4-m gait speed test and grip strength tests were updated to align with the National Institutes of Health toolbox.^29^

**Table 1:** Modified Fried's Frailty Phenotype

Updated frailty phenotype
Shrinking • >10 lb or >5% of body weight unintentional weight loss in prior year or BMI <18.5 kg/m^2^
Weakness • Lowest 20% grip strength by gender and BMI or inability to complete grip test. Best hand grip of 3 trials.
Exhaustion • Self-reported “exhaustion” based on the CES-D Depression scale “How often in the last week did you feel this way?” with response of either a “moderate amount of the time” or “most of the time” to: “I felt that everything I did was an effort.” “I could not get going.”
Slowness • Slowest 20% walking time/4 m by gender and height or inability to complete walking test. Static start, dynamic stop, faster of 2 walks.
Low activity • Lowest 20% Kcals/wk based on the modified Minnesota Leisure Time Activity Questionnaire^27^^,^^28^ asking about walking, strenuous chores, mowing the lawn, gardening, general exercise, golf. • Male <148 Kcals/wk • Female <105 Kcals/wk

A point is earned for meeting each of the following 5 criteria resulting in a score from 0 to 5. Not frail indicates 0 criteria present. Prefrail indicates 1 or 2 criteria present. Frail indicates 3 or more criteria present. Red text denotes changes from the original FFP.^1^*BMI,* Body mass index; *CES-D,* Center for Epidemiologic Studies Depression Scale.

**Figure E1:**
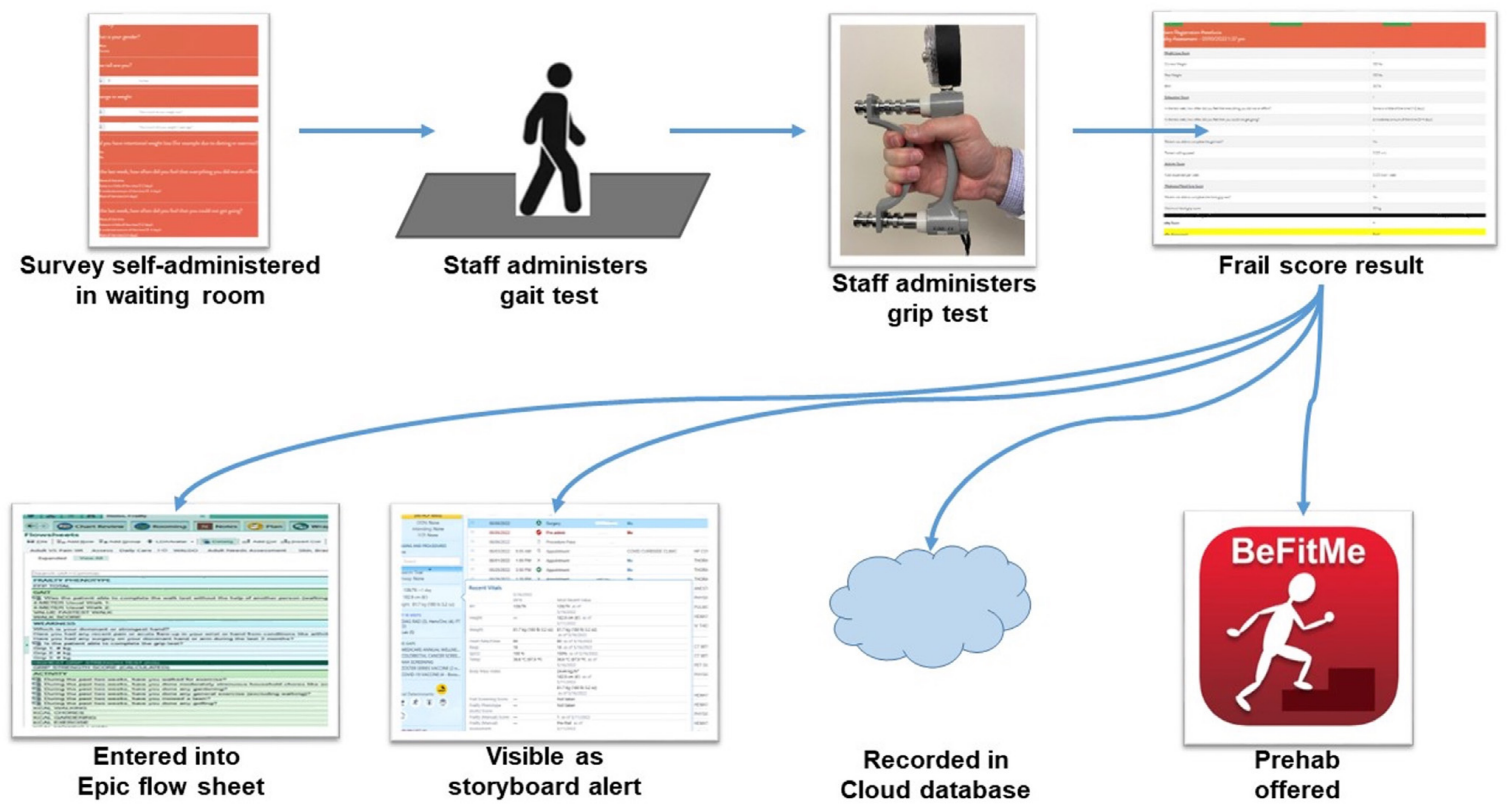
Frailty clinic screening workflow with the BeFitMe.

A webapp (BeFitMe) was designed to facilitate easy, comprehensive, and standardized frailty screening. While in the waiting room, patients filled out FFP survey questions on an electronic tablet to assess activity, exhaustion, and weight change (Table 1 and Figure E1). Clinic staff then administered the grip strength test using a hydraulic dynamometer (Jamar Hydraulic Hand Dynamometer, Sammons Preston) and recorded the best of 3 trials by the dominant hand on BeFitMe. Clinic staff then administered a 4-m usual gait speed test with a static start and dynamic stop; the faster walk of 2 walks was used to calculate the FFP score. Patients are only instructed specifically about the meaning of the testing if they ask, at which point staff inform them: “We screen every new patient who is aged more than 50 years to get an understanding of their current physical condition.” BeFitMe awards a single point each time a patients meets criteria for any of the 5 components to tabulate a FFP score ranging from 0 to 5 with patients designated as not frail (0), prefrail (1-2), or frail (3-5). Results were then entered into the electronic medical record (Figure E1).

### Perioperative Outcomes

For screened patients who underwent surgery, perioperative outcomes were reviewed, including length of stay, discharge to location other than home, respiratory complications (prolonged air leak, pleural effusion requiring drainage, pneumonia, acute respiratory distress syndrome, respiratory failure requiring reintubation, pneumothorax, prolonged postoperative ventilatory support, or tracheostomy), cardiac complications (myocardial infarction, arrythmia requiring intervention), any complication (respiratory complications, cardiac complications, cerebrovascular accident, pulmonary embolism, hyperglycemic episode, urinary tract infection, surgical site infection, sepsis, unplanned intensive care unit admission, or unexpected return to the operating room), 90-day readmission, and 90-day mortality (Table 6).

### Statistical Analysis

The primary outcomes of this study were frailty status and the FFP score. Means (continuous) and frequencies (categorical) were compared for patient characteristics of subgroups differentiated by frailty and race. Statistical significance of differences between racial groups was assessed by chi-square test for categorical variables and Student *t* test for continuous variables. The association of race and FFP score was evaluated with univariate ordinal regression. Logistic regressions performed with binomial distribution further assessed the association of patient demographic and clinical characteristics with various frailty statuses. Regression models investigated associations between race and being categorized as (a) frail and prefrail versus not frail; (b) frail versus not frail; and (c) prefrail versus not frail. Models were constructed with 3 levels: (1) crude (univariate) analysis of racial differences; (2) multivariate analysis including covariates of patient race, age, sex, median income, BMI category, and total number of comorbidities; and (3) multivariate analysis that added pulmonary function tests (FEV_1_ and DLCO as a percentage of the predicted value for that patient) to the prior multivariate model for the subset of patients for whom these data were available. Covariates were identified as factors associated with increased incidence of frailty in prior analyses.^14^, ^15^, ^16^^,^^18^, ^19^, ^20^ Models were reported with calculated odds ratios (ORs) and 95% CIs. *P* values for both logistic and ordinal regression were assessed via Wald's test. Statistical analysis was performed in R, version 4.2.0 (Foundation for Statistical Computing).

## Results

### Participants

Between December 2020 and June 2022, 317 patients were screened for frailty, of whom 43.5% underwent surgery. The majority of patients presented for evaluation of lung nodules (65.8%) (Table 2). Most patients were NHW, female, and either current or former smokers. Pulmonary function tests (FEV1 and DLCO) were available for 162 patients (51%) screened. Of participants screened, 18.6% were frail, 43.8% were prefrail, and 37.5% were not frail. A minority (36/317) of patients did not fit into either the NHB or NHW categories. These patients self-identified as Asian (9), Native American (3), Hispanic White (7), Other (5), or preferred not to specify (12). These patients were excluded from analyses directly comparing NHB and NHW populations.

**Table 2:** Patient demographics

Demographics	Overall (N = 317)	NHB (N = 106)	NHW (N = 175)	*P* value
Age	68.1 (8.60)	67.4 (8.21)	68.7 (8.57)	.204
Female	187 (59.0%)	65 (61.3%)	98 (56.0%)	.453
Marital status				
Single/never married	62 (19.6%)	48 (45.3%)	12 (6.9%)	<.001∗∗∗
Domestic partner	3 (0.9%)	0 (0%)	3 (1.7%)	
Married	183 (57.7%)	32 (30.2%)	127 (72.6%)	
Separated	2 (0.6%)	2 (1.9%)	0 (0%)	
Divorced	27 (8.5%)	11 (10.4%)	15 (8.6%)	
Widow/widower	29 (9.1%)	9 (8.5%)	14 (8.0%)	
Unknown	11 (3.5%)	4 (3.8%)	4 (2.3%)	
Median income	$67,400 ($26,500)	$46,500 ($15,000)	$78,400 ($24,300)	<.001∗∗∗
First quartile (<$44,700)	84 (26.5%)	71 (67.0%)	9 (5.1%)	<.001∗∗∗
Second quartile ($44,701-63,200)	75 (23.7%)	18 (17.0%)	50 (28.6%)	
Third quartile ($63,201-84,100)	79 (24.9%)	15 (14.2%)	51 (29.1%)	
Fourth quartile (>$84,101)	79 (24.9%)	2 (1.9%)	65 (37.1%)	
BMI				
Underweight	14 (4.4%)	5 (4.7%)	9 (5.1%)	.154
Normal weight	99 (31.2%)	27 (25.5%)	56 (32.0%)	
Overweight	101 (31.9%)	29 (27.4%)	59 (33.7%)	
Obese	103 (32.5%)	45 (42.5%)	51 (29.1%)	
Smoker status				
Current smoker	69 (21.8%)	38 (35.8%)	29 (16.6%)	.002∗∗
Former smoker	151 (47.6%)	46 (43.4%)	91 (52.0%)	
Never smoker	96 (30.3%)	22 (20.8%)	54 (30.9%)	
No of comorbidities	2.09 (1.37)	2.56 (1.45)	1.91 (1.28)	<.001∗∗∗
0	113 (35.6%)	23 (21.7%)	73 (41.7%)	<.001∗∗∗
1-2	152 (47.9%)	56 (52.8%)	80 (45.7%)	
3+	52 (16.4%)	27 (25.5%)	22 (12.6%)	
DM	75 (23.7%)	32 (30.2%)	33 (18.9%)	.042∗
HTN	198 (62.5%)	81 (76.4%)	97 (55.4%)	<.001∗∗∗
CAD	36 (11.4%)	10 (9.4%)	23 (13.1%)	.456
History of MI	10 (3.2%)	3 (2.8%)	6 (3.4%)	1
CHF	21 (6.6%)	14 (13.2%)	7 (4.0%)	.009∗∗
DVT	14 (4.4%)	5 (4.7%)	9 (5.1%)	1
Asthma	28 (8.8%)	18 (17.0%)	9 (5.1%)	.002∗∗
COPD	55 (17.4%)	23 (21.7%)	30 (17.1%)	.43
CKD	19 (6.0%)	11 (10.4%)	7 (4.0%)	.062
Arthritis	53 (16.7%)	22 (20.8%)	28 (16.0%)	.396
History of cancer	127 (40.1%)	38 (35.8%)	78 (44.6%)	.241
Pulmonary function tests				
DLCO%	75.2 (26.9)	70.8 (28.1)	76.7 (27.0)	.213
FEV1%	80.3 (26.2)	76.2 (24.6)	82.3 (27.9)	.154
New cancer diagnosis	139 (43.8%)	39 (36.8%)	80 (45.7%)	.264
Reason for evaluation				
Esophagus-benign	3 (1.1%)	1 (0.9%)	2 (1.1%)	.026
Esophagus-malignant	10 (3.6%)	0 (0%)	10 (5.7%)	
Lung nodule-benign	2 (0.7%)	2 (1.9%)	0 (0%)	
Lung nodule-malignant	77 (27.4%)	28 (26.4%)	49 (28.0%)	
Lung nodule-unknown	106 (37.7%)	49 (46.2%)	57 (32.6%)	
Mediastinal mass	20 (7.1%)	7 (6.6%)	13 (7.4%)	
Other	63 (22.4%)	19 (17.9%)	44 (25.1%)	
Had operative intervention	138 (43.5%)	43 (40.6%)	81 (46.3%)	.396

Data presented as n (%) for categorical variables and mean (SD) for continuous variables. *P* values evaluated by chi-square test for categorical variables and Student *t* test for continuous variables. *NHB,* Non-Hispanic Black; *NHW,* non-Hispanic White; *BMI,* body mass index; *DM,* diabetes mellitus; *HTN,* hypertension; *CAD,* coronary artery disease; *MI,* myocardial infarction; *CHF,* congestive heart failure; *DVT,* deep vein thrombosis; *COPD,* chronic obstructive pulmonary disease; *CKD,* chronic kidney disease; *DLCO*, diffusion capacity of the lungs for carbon monoxide; *FEV1,* forced expiratory volume during 1 second. ∗*P* value <.05; ∗∗*P* value <.01; ∗∗∗*P* value <.001.

Compared with NHW patients, NHB patients were less likely to be married and were more likely to be current or former smokers (Table 2). In an analysis of patient income status, NHB patients came from areas with lower median income., and were more frequently from regions in the first quartile. NHW patients were more likely to have malignant esophageal or lung lesions as a visit diagnosis, whereas NHB patients were more likely to have unbiopsied (unknown) lung lesion. NHB patients also had a higher number of comorbidities compared with NHW patients, including higher prevalence of asthma, congestive heart failure, hypertension, diabetes, and chronic kidney disease. There were no differences in age, sex, or BMI between NHB and NHW patients. NHB patients had higher rates of frail status (23.6% vs 16.0%) and prefrail status (51.9% vs 39.4%) compared with NHW patients (Table 3).

**Table 3:** Patient Fried's frailty assessments by race

	NHB (N = 106)	NHW (N = 175)	*P* value
Frailty assessment			
Frail	25 (23.6%)	29 (16.0%)	.003∗∗
Prefrail	55 (51.9%)	71 (39.2%)	
Not frail	26 (24.5%)	81 (44.8%)	
Frailty component			
Gait score	40.0%	24.3%	.007∗∗
Shrinkage score	34.3%	23.8%	.063
Weakness score	26.7%	22.1%	.392
Exhaustion score	31.4%	26.5%	.383
Activity score	24.8%	14.4%	.038∗

Data presented as mean (SD) for continuous and n (%) for categorical variables. *NHB,* Non-Hispanic Black; *NHW,* non-Hispanic White. ∗*P* < .05; ∗∗*P* < .01; ∗∗∗*P* < .001.

### Factors Associated With Frailty Status

Compared with patients who were not frail, frail patients were older and more likely to be single or never married, had lower median income, had lower percent predicted DLCO, had lower percent predicted FEV1, and were less likely to undergo surgery (Table 4). NHB race significantly predicted overall FFP score by univariate ordinal regression (OR, 2.10, *P* < .001). NHB patients were more likely to be frail or prefrail than not frail when compared with NHW patients when performing multivariate logistic regression with binomial distribution both without (OR, 3.42; 95% CI, 1.59-7.37; *P* = .002) and with controlling for pulmonary function testing (OR, 3.77; 95% CI, 1.25-11.40; *P* = .019) (Table 5). In subset analyses, NHB patients were also more likely to be frail when excluding prefrail patients (Table E1).

**Table 4:** Demographic comparison between frail and not frail patients

Demographics	Not frail (N = 119)	Frail (N = 59)	*P* value
Age	67.2 (8.41)	71.0 (7.99)	.004∗∗
Sex (female)	64 (53.8%)	37 (62.7%)	.331
Marital status			
Single/never married	17 (14.3%)	16 (27.1%)	.011∗
Married	80 (67.2%)	27 (45.8%)	
Domestic partner	0 (0%)	1 (1.7%)	
Divorced	12 (10.1%)	3 (5.1%)	
Widow/widower	8 (6.7%)	8 (13.6%)	
Unknown	2 (1.7%)	4 (6.8%)	
Median income	$70,900 ($25,900)	$61,700 ($24,700)	.023∗
First quartile (<$44,700)	25 (21.0%)	19 (32.2%)	.123
Second quartile ($44,701-63,200)	25 (21.0%)	17 (28.8%)	
Third quartile ($63,201-84,100)	39 (32.8%)	13 (22.0%)	
Fourth quartile (>$84,101)	30 (25.2%)	10 (16.9%)	
BMI			
Underweight	1 (0.84%)	5 (8.47%)	.057
Normal weight	38 (31.9%)	20 (33.9%)	
Overweight	42 (35.3%)	19 (32.2%)	
Obese	38 (31.9%)	15 (25.4%)	
Smoker status			
Current smoker	19 (16.0%)	15 (25.4%)	.305
Former smoker	59 (49.6%)	30 (50.8%)	
Never smoker	40 (33.6%)	14 (23.7%)	
No of comorbidities	1.90 (1.17)	2.19 (1.36)	.168
0	46 (38.7%)	18 (30.5%)	.169
1-2	62 (52.1%)	30 (50.8%	
3+	11 (9.24%)	11 (18.6%)	
DM	22 (18.5%)	19 (32.2%)	.063
HTN	70 (58.8%)	38 (64.4%)	.579
CAD	9 (7.6%)	10 (16.9%)	.099
History of MI	5 (4.2%)	2 (3.4%)	1
CHF	5 (4.2%)	5 (8.5%)	.412
DVT	6 (5.0%)	5 (8.5%)	.572
Asthma	9 (7.6%)	4 (6.8%)	1
COPD	15 (12.6%)	12 (20.3%)	.258
CKD	3 (2.5%)	4 (6.8%)	.334
Arthritis	16 (13.4%)	8 (13.6%)	1
History of cancer	58 (48.7%)	17 (28.8%)	.039∗
Pulmonary function tests			
DLCO% (N = 84)	78.7 (24.6)	61.8 (26.1)	.006∗∗
FEV1% (N = 84)	86.0 (25.1)	73.9 (26.3)	.04∗
New cancer diagnosis	53 (44.5%)	25 (42.4%)	.666
Had operative intervention	62 (53%)	15 (25.9%)	.001∗∗

Data presented as n (%) for categorical variables and mean (SD) for continuous variables. *P* values evaluated by chi-square test for categorical variables and Student *t* test for continuous variables. *BMI,* Body mass index; *DM,* diabetes mellitus; *HTN,* hypertension; *CAD,* coronary artery disease; *MI,* myocardial infarction; *CHF,* congestive heart failure; *DVT,* deep vein thrombosis; *COPD,* chronic obstructive pulmonary disease; *CKD,* chronic kidney disease; *DLCO,* diffusion capacity of the lungs for carbon monoxide; *FEV1,* forced expiratory volume during 1 second. ∗*P* < .05; ∗∗*P* < .01; ∗∗∗*P* < .001.

**Table 5:** Logistic regression with binomial distribution evaluating odds of any frailty (frail status and prefrail) versus not frail status among thoracic surgery patients based on patient race (Model 1); previously associated demographic factors (Model 2); and pulmonary function tests (Model 3)

	Model 1 (crude) (N = 317)		Model 2 (multivariate) (N = 317)		Model 3 (including PFTs) N = (134)	
	OR (95% CI)	Wald's test (*P* value)	OR (95% CI)	Wald's test (*P* value)	OR (95% CI)	Wald's test (*P* value)
Race						
White (ref)	1 (1-1)	-	1 (1-1)	-	1 (1-1)	
Black	2.47 (1.45-4.22)	<.001∗∗∗	3.42 (1.59-7.37)	.002∗∗	3.77 (1.25 -11.40)	.019∗
Other	1.13 (0.54-2.33)	.749	1.35 (0.63-2.88)	.444	1.45 (0.44-4.83)	.543
Age			1.02 (0.99-1.05)	.131	1.05 (1.00-1.11)	.059
Sex (male)			0.74 (0.45-1.23)	.247	0.53 (0.24-1.16)	.112
Income Quartile						
First (ref)			1 (1-1)	-	1 (1-1)	-
Second			1.78 (0.75-4.22)	.188	0.59 (0.16-2.21)	.433
Third			1.03 (0.45-2.38)	.939	0.42 (0.11-1.60)	.205
Fourth			2.06 (0.83-5.10)	.120	0.68 (0.16-2.92)	.606
BMI Category						
Underweight			9.03 (1.09-74.98)	.042∗	8.81 (0.82-94.52)	.072
Normal (ref)			1 (1-1)	-	1 (1-1)	-
Overweight			0.96 (0.53-1.77)	.907	2.33 (0.87-6.25)	.093
Obese			0.92 (0.50-1.69)	.790	1.32 (0.53-3.28)	.553
No of comorbidities						
0 (ref)			1 (1-1)	-	1 (1-1)	-
1-2			0.90 (0.52-1.56)	.712	0.74 (0.32-1.70)	.472
3+			2.23 (0.96-5.18)	.061	1.61 (0.49-5.22)	.431
Pulmonary function tests						
DLCO % of predicted					1.01 (0.99-1.03)	.323
FEV1 % of predicted					0.99 (0.97-1.00)	.172

Ref is the categorical variable used as a reference for comparisons. *PFTs*, Pulmonary function tests; *OR,* odds ratio; *CI*, confidence interval; *BMI,* body mass index; *DLCO,* diffusion capacity of the lungs for carbon monoxide; *FEV1,* forced expiratory volume during 1 second. ∗*P* < .05; ∗∗*P* < .01; ∗∗∗*P* < .001.

Factors associated with prefrail status were also assessed. Other than race and having 3+ comorbid conditions, there were no patient characteristics that were significantly associated with patients assessed as prefrail when compared with not frail (Table E2). NHB patients were more likely to be prefrail than not frail when compared with NHW patients (Table E3).

### Factors Associated With Fried's Frailty Phenotype Score Components

When analyzing components of the FFP, NHB patients were more likely to have slow gait (40% vs 24%, *P* = .007) and low activity (25% vs 14%, *P* = .05) than NHW patients (Table 3). NHB patients also trended toward being more likely to score a point for shrinkage than NHW patients.

### Postoperative Outcomes for Frail Patients

Of the 138 patients who underwent surgery, 15 (10.9%) were frail and 61 (44.2%) were prefrail. When compared with not frail patients, frail patients were less likely to be discharged home, had higher 90-day perioperative mortality, had higher 90-day readmission rates, and trended toward higher rates of cardiac complications, respiratory complications, any perioperative complication, and longer length of stay (Table 6). No differences in outcomes were seen between prefrail and not frail patients, or between NHB and NHW patients.

**Table 6:** Perioperative outcomes for frail and prefrail patients when compared with not frail patients

Outcomes	Not frail (N = 62)		Prefrail (N = 61)		Frail (N = 15)	
	N (%)	*P* value	N (%)	*P* value	N (%)	*P* value
Perioperative complications						
Cardiac	7 (11.3%)	-	6 (9.8%) 1	1	3 (20%)	.637
Respiratory	11 (17.7%)	-	17 (27.9%)	0.261	5 (33.3%)	.327
Any	18 (29.0%)	-	26 (42.6%)	0.166	8 (53.3%)	.138
Length of stay	3.0 [0-15]	-	3.0 [0-14]	0.342	5.5 [0-31]	.105
Discharge to home	60 (96.8%)	-	56 (91.8%)	0.312	11 (73.3%)	.01
90-d readmission	4 (6.5%)	-	11 (18.0%)	0.092	7 (46.7%)	<.001
90-d mortality	0 (0%)	-	1 (1.6%)	0.993	5 (33.3%)	<.001

## Discussion

**Figure 1:**
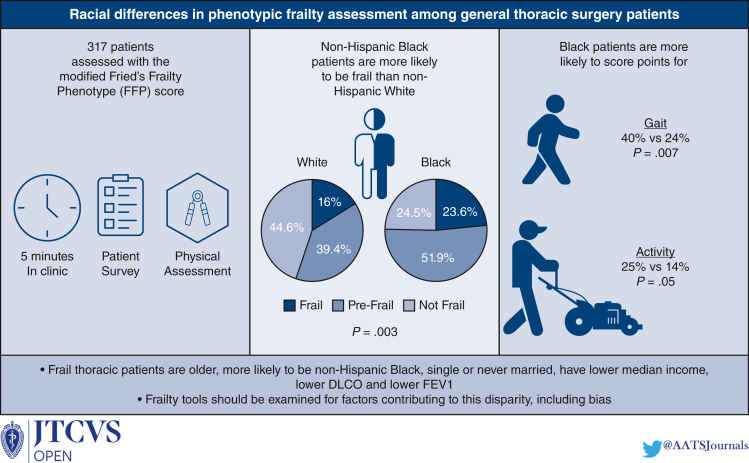
Graphical Abstract. *DLCO,* Diffusion capacity of the lungs for carbon monoxide; *FEV1,* forced expiratory volume in 1 second.

Given the American Association for Thoracic Surgery recent emphasis on the importance of frailty in the assessment of the patient undergoing thoracic surgery, we analyzed our protocol for frailty evaluation and the factors associated with frailty as determined by FFP in our patients undergoing thoracic surgery. All patients presenting for surgical evaluation were routinely screened for frailty to ascertain their perioperative risk and inform their treatment recommendations. Consistent with studies evaluating the general population, we found that frail patients undergoing thoracic surgery were older,^17^ were more likely to be single or never married,^14^^,^^18^ and had lower median income,^18^^,^^20^ percent predicted DLCO, and percent predicted FEV1.^15^, ^16^, ^17^ We also evaluated racial differences in frailty assessment among general thoracic surgery patients and found that NHB patients were more likely to be frail and score higher on the FFP than NHW patients. These results were driven by differences in activity level and gait speed, and persisted when controlling for comorbidity burden and demographic factors, consistent with National Health and Aging Trends Study data (Figure 1).^23^

It has been shown that the average gait speed of NHB patients is lower than that of NHW patients, and decreased gait speed is associated with increased mortality among patients undergoing cardiothoracic surgery.^13^^,^^21^^,^^23^^,^^30^ The etiology of this gait difference could be physiologic or cultural,^30^, ^31^ but how gait speed contributes to surgical morbidity and mortality is not clear. Because frailty assessments such as the FFP rely on volitional gait speed, and volitional gait speed is lower on average for NHB patients,^29^ then these frailty assessments may not purely identify frailty status but also reflect race. Other frailty assessment tools that do not incorporate gait speed, such as the FRAIL scale developed from middle-aged African American patients living in St Louis, have not demonstrated racial differences in frailty prevalence and may have similar predictive ability for postoperative complications.^32^^,^^33^

Low activity level was also more frequently seen in our NHB patients compared with NHW patients. This difference in activity level has not been observed in prior work looking at differences by race and ethnicity.^23^ Difference in activity level may reflect the nature of the activity choices presented to patients (Table 1). Patients living in an urban environment may have less opportunity to engage in activities such as gardening, golfing, or mowing their lawn than those in more suburban or rural environments. This difference in activity level also could be due to lack of safe outdoor spaces in the predominantly NHB neighborhoods on the South Side of Chicago that the University of Chicago serves, which has one of the highest gun-violence rates in the country.^34^

Racial disparities in frailty status also may be a physiologic manifestation of differences in social determinants of health and the lived experience of these populations. In this study, NHB patients and frail patients were both more likely to have lower median income and less likely to be married. Frailty is most often defined as a diminished physiologic reserve that makes an individual more vulnerable to a stressor.^1^ Racial discrimination against NHB patients can contribute to increased allostatic load, which is associated with diminished physiologic reserve and increased rates of physiologic frailty.^35^^,^^36^ NHB patients also more frequently presented with an unbiopsied lung nodule, which may represent disparate access to care relative to NHW patients, further emphasizing the social vulnerability of this population. More work is needed to evaluate the impact of the social environment on physiologic frailty.

The majority of NHB patients (51%) in our population were assessed as prefrail. The clinical implications of prefrailty status have not been studied as extensively as frailty status, but recent data show that patients identified as prefrail by the FFP have higher 1-year mortality than nonfrail patients among Medicare beneficiaries.^37^

Frailty assessment tools range widely.^10^^,^^38^ Some are based on retrospective compilation of comorbidities (Canada Study of Health and Aging Frailty Index and its derivatives the modified 5-item and 11-item Frailty Indices; the administrative Risk Analysis Index), others on self-reported questionnaires (FRAIL scale; the clinical Risk Analysis Index), or clinician impression (Rockwood's Clinical Frail Scale), and some incorporate objective functional measurements (FFP; Edmonton Frail Scale).^1^^,^^23^^,^^32^^,^^39^, ^40^, ^41^, ^42^, ^43^ Some groups simplify the frailty evaluation process by using single measures to detect frailty, such as the timed-up-and-go test or 4-m usual walk test.^13^^,^^21^ Our group believes the combination of patient-reported symptoms and physical assessment used in FFP provides the best available measurement of frailty at the time of surgical evaluation.

Neither comorbidity burden nor age was associated with increased frailty in our multivariate analysis. This study may be underpowered to find age as a statistically significant variable with its use as a continuous as opposed to categorical variable. There was a trend toward higher comorbidity burden among frail patients, but perhaps because FFP does not base its criteria on comorbidity burden, it is identifying physiologic vulnerability regardless of the presence of multimorbidity.

### Study Limitations

This study has several limitations. We were not able to study racial discrepancies in other historically marginalized populations, namely, Asian and Hispanic patients, due to small patient population. This study also determined patient socioeconomic status using median income as derived from residential ZIP code, which may not accurately reflect individual income. We were unable to make strong assertions on the effect of frailty on surgical outcomes at this time based on the relatively few (138, 43.5%) patients who underwent surgery in this cohort, although that is an area of ongoing research. Last, our current study only evaluated 1 measurement of frailty, the FFP. Although FFP is the most cited metric assessing for physiologic frailty, in part because it combines patient subjective experience and symptoms with objective assessments of their physical fitness, a gold standard for frailty measurement does not exist.^10^ Although other frailty metrics have been validated in more diverse populations including the FRAIL scale, the popularity of the FFP and gait speed as a single measure of frailty warrant increased scrutiny of the unequal racial distribution of frailty as assessed by FFP.

## Conclusions

NHB patients undergoing general thoracic surgery are more likely to have higher FFP scores and be categorized as frail or prefrail than NHW patients. This disparity is related to differences in activity and gait speed. Racial differences in frailty measurements are not adequately adjusted for when controlling for age, sex, BMI, median income, and comorbid status. These findings draw into question whether the scoring system devised by Fried and colleagues^1^ is accurately measuring frailty in all populations, suggesting the possibility of intrinsic and institutional bias. Understanding factors associated with frailty status provides opportunities to identify and mitigate disparities during surgical evaluation and treatment.

## Conflict of Interest Statement

The authors reported no conflicts of interest.

The *Journal* policy requires editors and reviewers to disclose conflicts of interest and to decline handling or reviewing manuscripts for which they may have a conflict of interest. The editors and reviewers of this article have no conflicts of interest.

## Figures and Tables

**Table E1:** Logistic regression with binomial distribution evaluating odds of frail status versus not frail status among thoracic surgery recipients based on patient race (Model 1); previously associated demographic factors (Model 2); and pulmonary function tests (Model 3)

	Model 1 (crude) (N = 178)		Model 2 (multivariate) (N = 178)		Model 3 (including PFTs) N = (162)	
	OR (95% CI)	Wald's test (*P* value)	OR (95% CI)	Wald's test (*P* value)	OR(95% CI)	Wald's test (*P* value)
Race						
White (ref)	1 (1-1)	-	1 (1-1)	-	1 (1-1)	
Black	2.68 (1.33-5.39)	.006∗∗	2.79 (1.03-7.58)	.044∗	5.81 (1.27-29.92)	.035∗
Other	1.11 (0.39-3.15)	.838	1.29 (0.41-3.98)	.664	0.92 (0.07-13.03)	.952
Age			1.05 (1.01-1.10)	.013∗	1.06 (0.96-1.17)	.247
Sex (male)			0.80 (0.38-1.67)	.553	0.37 (0.09-1.55	.175
Income quartile						
First (ref)			1 (1-1)	-	1 (1-1)	-
Second			1.63 (0.54-4.97)	.388	0.43 (0.06-2.98)	.393
Third			1.03 (0.33-3.16)	.962	0.45 (0.07-2.93)	.401
Fourth			1.16 (0.33-4.10)	.819	0.48 (0.05-4.41)	.517
BMI Category						
Underweight			7.21 (0.73-71.54)	.091	5.99 (0.377-95.19)	.205
Normal (ref)			1 (1-1)	-	1 (1-1)	-
Overweight			1.07 (0.45-2.54)	.877	8.30 (1.39-49.62)	.020∗
Obese			0.67 (0.28-1.61)	.371	1.33 (0.26-6.93)	.733
No of comorbidities						
0 (ref)			1 (1-1)	-	1 (1-1)	-
1-2			1.18 (0.54-2.59)	.686	0.35 (0.08-1.55)	.167
3+			2.23 (0.73-6.80)	.160	1.49 (0.24-9.34)	.673
Pulmonary function tests						
DLCO % of predicted					0.98 (0.95-1.01)	.237
FEV1 % of predicted					0.99 (0.96-1.03)	.706

Ref is the categorical variable used as a reference for comparisons. *PFTs*, Pulmonary function tests; *OR,* odds ratio; *CI*, confidence interval; *BMI,* body mass index; *DLCO,* diffusion capacity of the lungs for carbon monoxide expressed as percent predicted; *FEV1,* forced expiratory volume in 1 second. ∗*P* < .05; ∗∗*P* < .01; ∗∗∗*P* < .001.

**Table E2:** Comparison of characteristics of prefrail and not frail patients

	Not frail (N = 119)	Prefrail (N = 139)	*P* value
Age	67.2 (8.41)	67.7 (8.80)	.686
Sex (female)	64 (53.8%)	86 (61.9%)	.236
Marital status			
Single/never married	17 (14.3%)	29 (20.9%)	.223
Married	80 (67.2%)	2 (1.4%)	
Domestic partner	0 (0%)	76 (54.7%)	
Separated	0 (0%)	2 (1.4%)	
Divorced	12 (10.1%)	12 (8.6%)	
Widow/widower	8 (6.7%)	13 (9.4%)	
Unknown	2 (1.7%)	5 (3.6%)	
Median income	$70,900 ($25,900)	$66,900 ($27,500)	.228
First quartile (<$44,700)	25 (21.0%)	40 (28.8%)	.093
Second quartile ($44,701-63,200)	25 (21.0%)	33 (23.7%)	
Third quartile ($63,201-84,100)	39 (32.8%)	27 (19.4%)	
Fourth quartile (>$84,101)	30 (25.2%)	39 (28.1%)	
BMI			
Underweight	1 (0.84%)	8 (5.76%)	.126
Normal weight	38 (31.9%)	41 (29.5%)	
Overweight	42 (35.3%)	40 (28.8%)	
Obese	38 (31.9%)	50 (36.0%)	
Smoker status			
Current smoker	19 (16.0%)	35 (25.2%)	.227
Former smoker	59 (49.6%)	62 (44.6%)	
Never smoker	40 (33.6%)	42 (30.2%)	
No of comorbidities	1.90 (1.17)	2.20 (1.52)	.073
0	46 (38.7%)	49 (35.3%)	.024∗
1-2	62 (52.1%)	60 (43.2%)	
3+	11 (9.24%)	30 (21.6%)	
DM	22 (18.5%)	34 (24.5%)	.313
HTN	70 (58.8%)	90 (64.7%)	.396
CAD	9 (7.6%)	17 (12.2%)	.301
History of MI	5 (4.2%)	3 (2.2%)	.559
CHF	5 (4.2%)	11 (7.9%)	.33
DVT	6 (5.0%)	3 (2.2%)	.359
Asthma	9 (7.6%)	15 (10.8%)	.5
COPD	15 (12.6%)	28 (20.1%)	.146
CKD	3 (2.5%)	12 (8.6%)	.068
Arthritis	16 (13.4%)	29 (20.9%)	.161
History of cancer	58 (48.7%)	52 (37.4%)	.15
Pulmonary function tests			
DLCO % predicted (N = 84)	78.7 (24.6)	77.5 (27.6)	.777
FEV1 % predicted (N = 88)	86.0 (25.1)	78.8 (26.4)	.105
New cancer diagnosis	53 (44.5%)	61 (43.9%)	.807
Had operative intervention	62 (53%)	61 (45.2%)	.267

Data presented as n (%) for categorical variables and mean (SD) for continuous variables. *P* values evaluated by chi-square test for categorical variables and Student *t* test for continuous variables. *BMI,* Body mass index; *DM,* diabetes mellitus; *HTN,* hypertension; *CAD,* coronary artery disease; *MI,* myocardial infarction; *CHF,* congestive heart failure; *DVT,* deep vein thrombosis; *COPD,* chronic obstructive pulmonary disease; *CKD,* chronic kidney disease; *DLCO,* diffusion capacity of the lungs for carbon monoxide; *FEV1,* forced expiratory volume in 1 second. ∗*P* < .05; ∗∗*P* < .01; ∗∗∗*P* < .001.

**Table E3:** Logistic regression with binomial distribution evaluating odds of prefrail versus frail status among thoracic surgery patients based on patient race (Model 1); previously associated demographic factors (Model 2); and pulmonary function tests (Model 3)

	Model 1 (crude) (N = 258)		Model 2 (multivariate) (N = 258)		Model 3 (including PFTs) N = (134)	
	OR (95% CI)	Wald's test (*P* value)	OR (95% CI)	Wald's test (*P* value)	OR (95% CI)	Wald's test (*P* value)
Race						
White (ref)	1 (1-1)	-	1 (1-1)	-	1 (1-1)	
Black	2.39 (1.36-4.22)	.003∗∗	3.76 (1.61-58.78)	.002∗∗	3.31 (0.97-11.27)	.056
Other	1.13 (0.52-2.48)	.838	1.38 (0.61-3.13)	.446	1.55 (0.45-5.43)	.489
Age			1.01 (0.98-1.04)	.583	1.06 (1.00-1.12)	.045∗
Sex (male)			0.76 (0.42-1.28)	.276	0.58 (0.25-1.34)	.206
Income quartile						
First (ref)			1 (1-1)	-	1 (1-1)	-
Second			1.77 (0.69-4.58)	.235	0.59 (0.14-2.45)	.467
Third			1.11 (0.4-2.83)	.824	0.43 (0.10-1.83)	.252
Fourth			2.74 (1.00-7.46)	.049	0.70 (0.14-3.44)	.661
BMI Category						
Underweight			8.99 (1.00-80.84)	.050∗	10.02 (0.79-126.44)	.075
Normal (ref)			1 (1-1)	-	1 (1-1)	-
Overweight			1.04 (0.53-2.04)	.903	1.70 (0.60-4.81)	.321
Obese			1.08 (0.56-2.09)	.824	1.30 (0.50-3.39)	.587
No of comorbidities						
0 (ref)			1 (1-1)	-	1 (1-1)	-
1-2			0.80 (0.44-1.46)	.468	0.84 (0.34-2.08)	.708
3+			2.28 (0.93-5.61)	.073	1.87 (0.53-6.54)	.330
Pulmonary function tests						
DLCO % of predicted					1.02 (1.00-1.04)	.113
FEV1 % of predicted					0.98 (0.96-1.00)	.105

Ref is the categorical variable used as a reference for comparisons. *PFTs*, Pulmonary function tests; *OR,* odds ratio; *CI*, confidence interval; *BMI,* body mass index; *DLCO,* diffusion capacity of the lungs for carbon monoxide; *FEV1,* forced expiratory volume in 1 second. ∗*P* < .05; ∗∗*P* < .01; ∗∗∗*P* < .001.
